# An mTOR and VEGFR inhibitor combination arrests a doxorubicin resistant lung metastatic osteosarcoma in a PDOX mouse model

**DOI:** 10.1038/s41598-021-87553-9

**Published:** 2021-04-21

**Authors:** Hiromichi Oshiro, Yasunori Tome, Kentaro Miyake, Takashi Higuchi, Norihiko Sugisawa, Fuminori Kanaya, Kotaro Nishida, Robert M. Hoffman

**Affiliations:** 1 AntiCancer Inc., 7917 Ostrow Street, San Diego, CA 92122 USA; 2 Department of Surgery, University of California, San Diego, CA USA; 3 Department of Orthopedic Surgery, Graduate School of Medicine, University of the Ryukyus, 207 Uehara, Nishihara-cho, Nakagami-gun, Okinawa, 903-0215 Japan

**Keywords:** Cancer, Medical research, Molecular medicine, Oncology

## Abstract

In order to identify more effective therapy for recalcitrant osteosarcoma, we evaluated the efficacy of an mTOR-VEGFR inhibitor combination on tumor growth in a unique osteosarcoma patient-derived orthotopic xenograft (PDOX) mouse model derived from the lung metastasis of an osteosarcoma patient who failed doxorubicin therapy. We also determined the efficacy of this inhibitor combination on angiogenesis using an in vivo Gelfoam fluorescence angiogenesis mouse model implanted with osteosarcoma patient-derived cells (OS-PDCs). PDOX models were randomly divided into five groups of seven nude mice. Group 1, control; Group 2, doxorubicin (DOX); Group 3, everolimus (EVE, an mTOR and VEGF inhibitor); Group 4, pazopanib (PAZ, a VEGFR inhibitor); Group 5, EVE-PAZ combination. Tumor volume and body weight were monitored 2 times a week. The in vivo Gelfoam fluorescence angiogenesis assay was performed with implanted OS-PDCs. The nude mice with implanted Gelfoam and OSPDCs also were divided into the four therapeutic groups and vessel length was monitored once a week. The EVE-PAZ combination suppressed tumor growth in the osteosarcoma PDOX model and decreased the vessel length ratio in the in vivo Gelfoam fluorescent angiogenesis model, compared with all other groups (p < 0.05). There was no significant body-weight loss in any group. Only the EVE-PAZ combination caused tumor necrosis. The present study demonstrates that a combination of an mTOR-VEGF inhibitor and a VEGFR inhibitor was effective for a DOX-resistant lung-metastatic osteosarcoma PDOX mouse model, at least in part due to strong anti-angiogenesis efficacy of the combination.

## Introduction

Osteosarcoma is a malignant bone sarcoma, which is most common in children and adolescents. Chemotherapy is first-line treatment of osteosarcoma along with surgery. First-line chemotherapy includes doxorubicin (DOX), methotrexate and cisplatinum. However, if first-line therapy fails, osteosarcoma becomes refractory and the number of second-line chemotherapy regimens is limited^1,2^. Therefore, new chemotherapy treatment strategies are needed for second- and third-line treatment of osteosarcoma.

A mammalian target of rapamycin (mTOR) inhibitor, everolimus (EVE), was approved as a treatment for advanced breast and renal cell cancer^3,4^. The mTOR inhibitor has anti-angiogenesis efficacy by suppressing the production of angiogenic factors in the mTOR pathway, which includes hypoxia-inducible factor 1α and vascular endothelial growth factor (VEGF)^5–8^.

A VEGF receptor (VEGFR) inhibitor, pazopanib (PAZ), was approved as a treatment for renal-cell cancer and soft-tissue sarcoma^9,10^. The VEGFR inhibitor has anti-angiogenesis efficacy by blocking blood-vessel formation^11–13^.

The efficacy of the combination of mTOR and VEGFR inhibitors was reported in pleural mesothelioma and urothelial carcinoma^14,15^. However, this combination has not been evaluated on osteosarcoma.

Our laboratory pioneered and established patient-derived orthotopic xenograft (PDOX) mouse models of the major cancers^16–22^ as well as many sarcoma types including osteosarcoma^23–27^. The PDOX mouse model mimics tumor characteristics of the patient, including metastases, compared with subcutaneous tumor mouse models^28^.

We have also established a fluorescent-protein based angiogenesis mouse model that can image angiogenesis induced by cancer cells in Gelfoam (Pfizer, New York, NY, U.S.A.) implanted in mice^29–32^. A previous study demonstrated strong angiogenesis by an osteosarcoma cell line using this model^31^.

In the present study, we assessed the efficacy of the combination of EVE and PAZ on a unique osteosarcoma PDOX mouse model derived from the lung metastasis of an osteosarcoma patient who failed DOX therapy. In addition, we assessed the efficacy of the EVE-PAZ combination on vessel length ratio in the in vivo Gelfoam angiogenesis mouse model implanted with osteosarcoma-patient-derived cells (OS-PDCs).

## Results

### Efficacy of the combination of EVE and PAZ on osteosarcoma PDOX tumor growth

**Figure 1:**
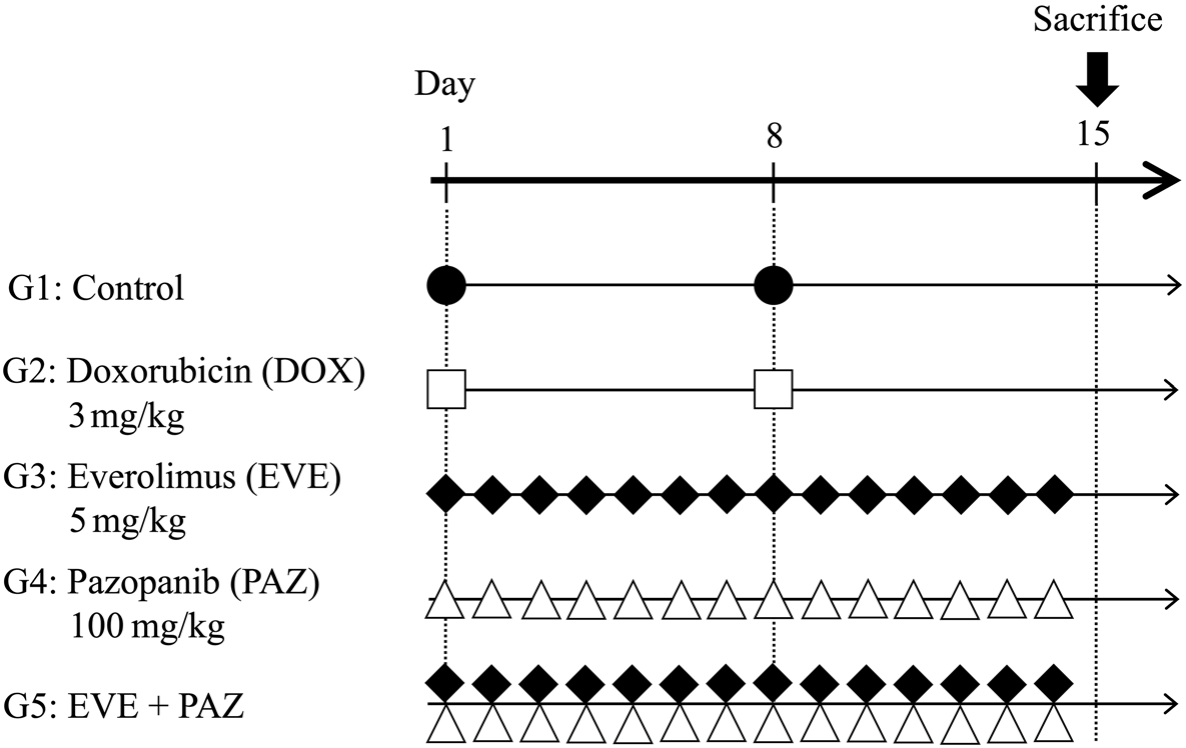
Treatment schema in an osteosarcoma PDOX model and in vivo Gelfoam angiogenesis model. Treatment started on day 1 and ended on day 15. Group 1, untreated control with PBS, i.p.; Group 2, DOX alone, 2.4 mg/kg, i.p., weekly for 2 weeks; Group 3, EVE alone, 3 mg/kg, oral gavage, daily for 2 weeks; Group 4, PAZ alone, 50 mg/kg, oral gavage, daily for 2 weeks; Group 5, treated with EVE and PAZ, 3 mg/kg and 50 mg/kg, respectively, oral gavage, daily for 2 weeks. Black circles, white squares, black squares and white triangles show administration schedule for each drug.

The therapeutic schedule for the DOX-resistant lung metastasis osteosarcoma PDOX mouse models is presented in Fig. 1. The treatment period was for 14 days.

**Figure 2:**
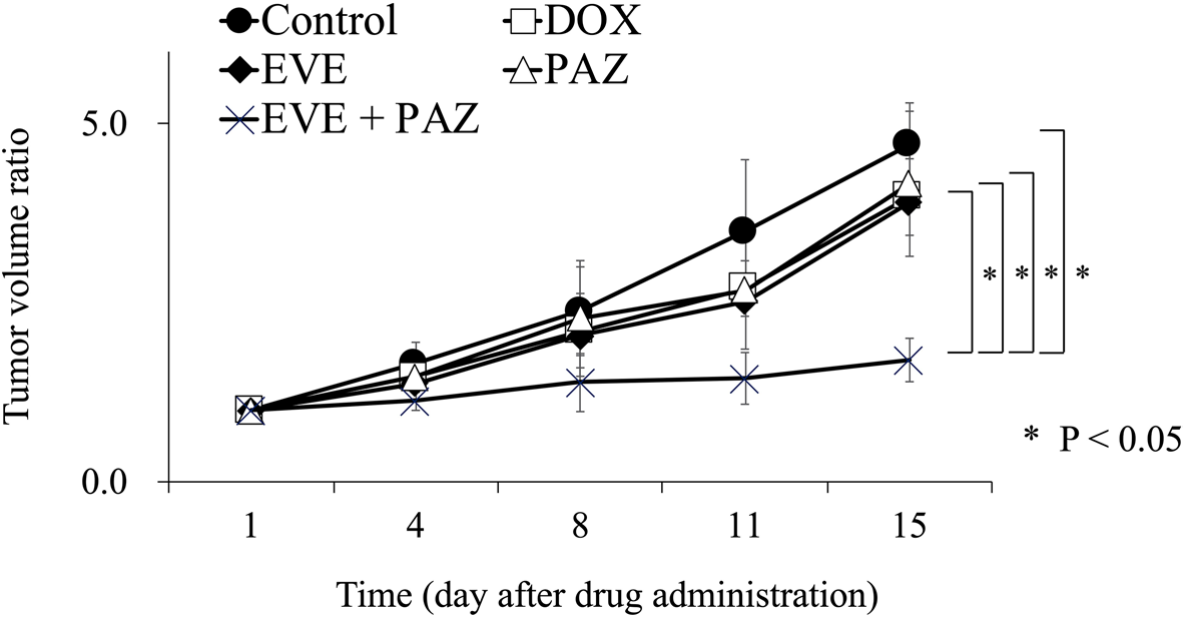
Efficacy of drugs on osteosarcoma growth in the PDOX mouse model. Line graphs express the tumor volume ratio, which is the tumor volume at any given time point relative to tumor volume at the beginning of the experiment. Line graphs express the average and error bars show ± standard deviation. Statistical analysis was performed using the Steel–Dwass test. *P < 0.05.

**Figure 3:**
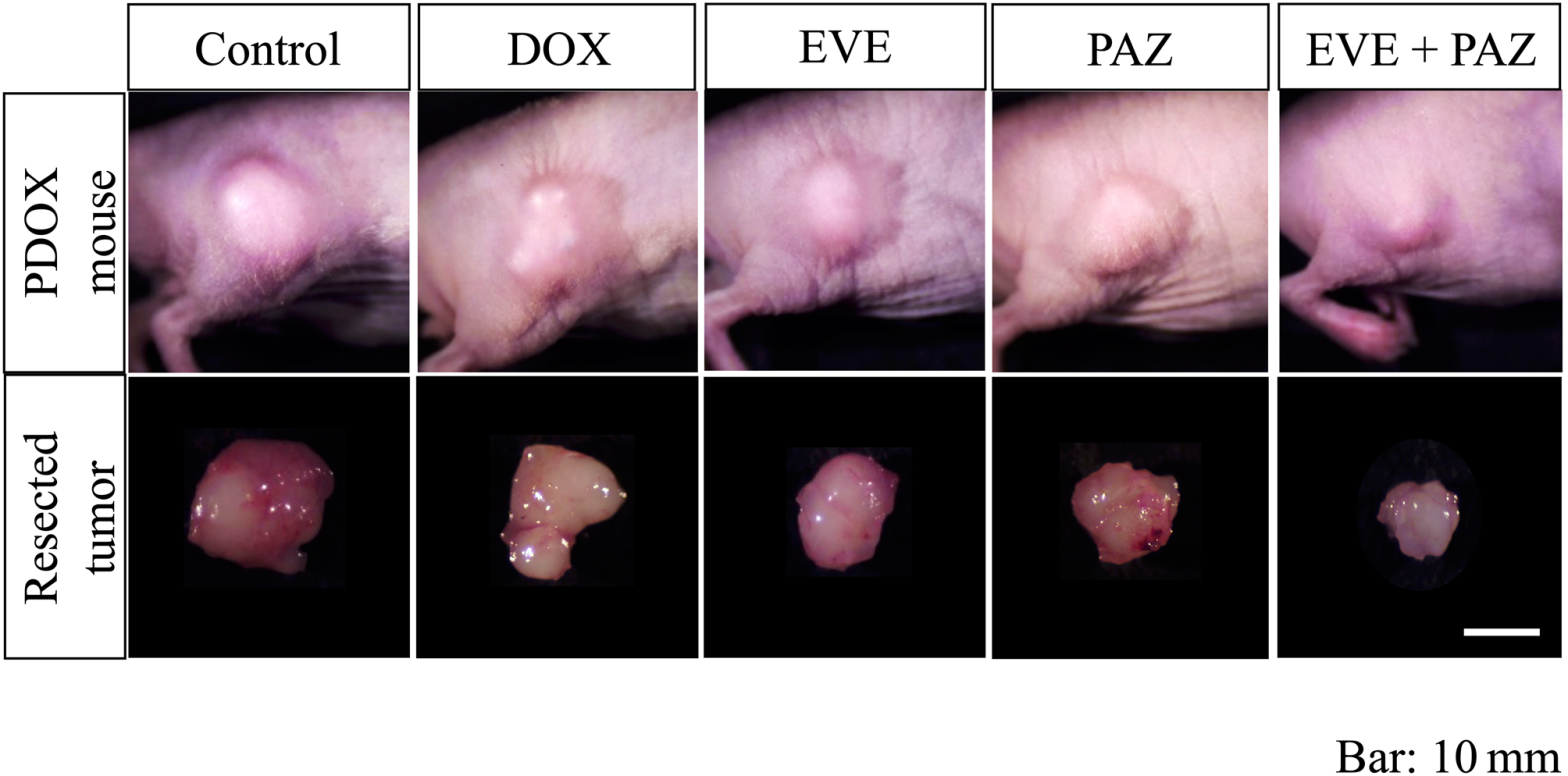
Photographs of control and treated in situ and resected tumors. The combination of EVE and PAZ inhibited osteosarcoma tumor growth in the PDOX model. Scale bars are 10 mm.

Tumor volume ratios at the end of the study were as follows: 4.70 ± 0.58 in Group 1 (untreated control group); 3.97 ± 0.53 in Group 2 (DOX); 3.89 ± 0.80 in Group 3 (EVE); 4.16 ± 1.01 in Group 4 (PAZ); and 1.70 ± 0.30 in Group 5 (EVE and PAZ). DOX, EVE-alone, and PAZ-alone showed no significant difference in the tumor-volume ratio compared with the untreated control group (p = 0.24, 0.38, 0.94, respectively). However, the combination of EVE and PAZ arrested tumor growth and had a significant difference in relative tumor volume compared with all other groups (p = 0.018) (Fig. 2). Representative photographs of tumors in situ and resected tumors also showed strong efficacy of the combination of EVE and PAZ (Fig. 3).

### Histopathology in the treated and untreated osteosarcoma PDOX mouse model

**Figure 4:**
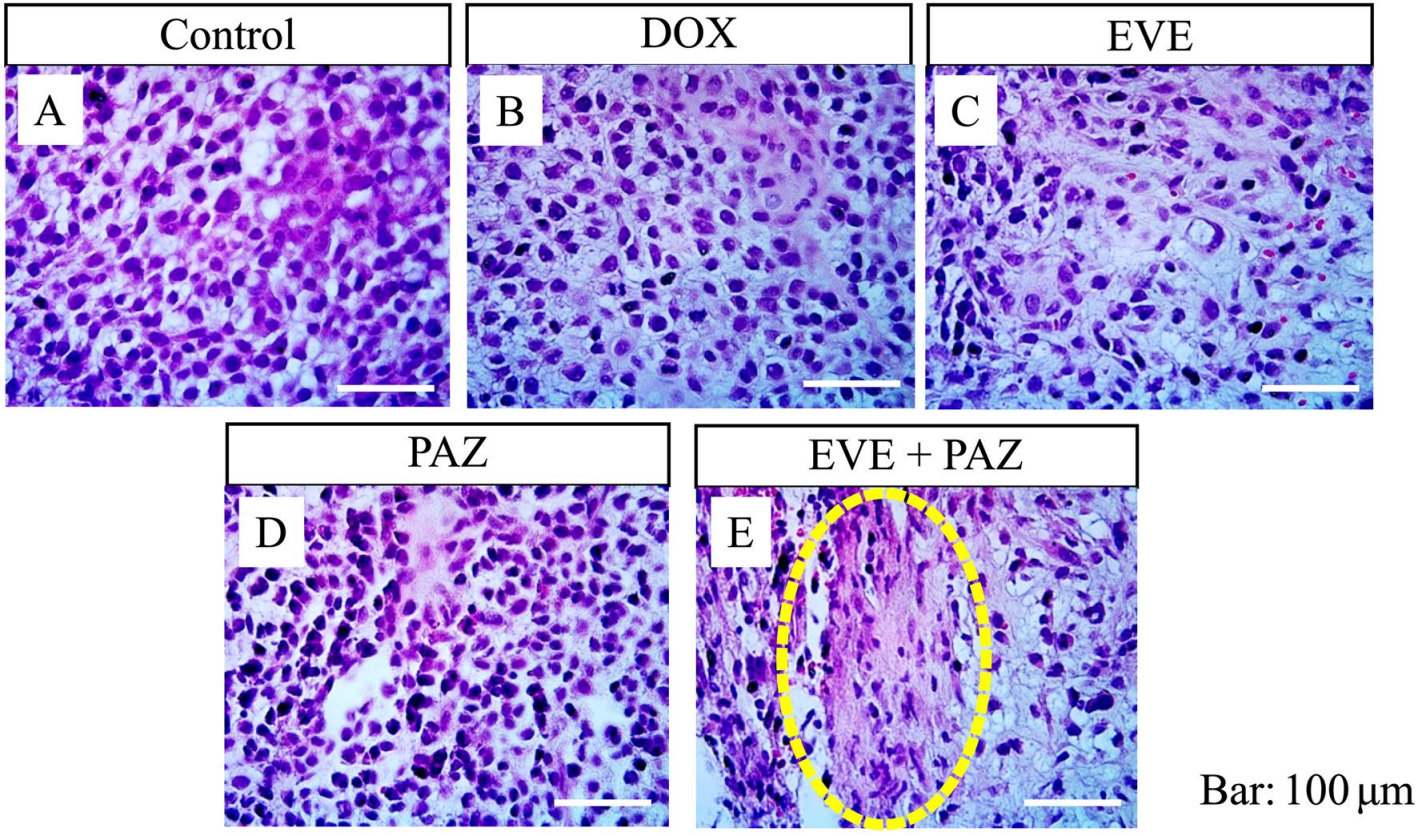
Efficacy of treatment of the osteosarcoma PDOX mouse model on tumor histopathology. Hematoxylin & eosin staining. (**A**) Group 1 treated with PBS; (**B**) Group 2 treated with DOX; (**C**) Group 3 treated with EVE-alone; (**D**) Group 4 treated with PAZ-alone; (**E**) Group 5 treated with the combination of EVE and PAZ. Yellow arrows indicate necrotic areas within the tumor. Scale bars are 100 μm.

The untreated control-group tumor had a high density of pleomorphic and spindle-shaped osteosarcoma cells. Osteosarcoma cells in the DOX, EVE-alone, and PAZ-alone groups had a slightly lower density compared with the untreated-control group. However, the combination of EVE and PAZ resulted in a lower density of osteosarcoma cells and had more necrosis compared with all other groups (Fig. 4).

### Body weight in the osteosarcoma PDOX mouse model

Animal deaths were not observed in any group. Body weight ratios at the end of the study were 1.05 ± 0.04 in Group 1 (untreated control); 1.04 ± 0.04 in Group 2 (DOX); 1.04 ± 0.06 in Group 3 (EVE); 1.04 ± 0.03 in Group 4 (PAZ); and 0.99 ± 0.05 in Group 5 (EVE and PAZ).

**Figure 5:**
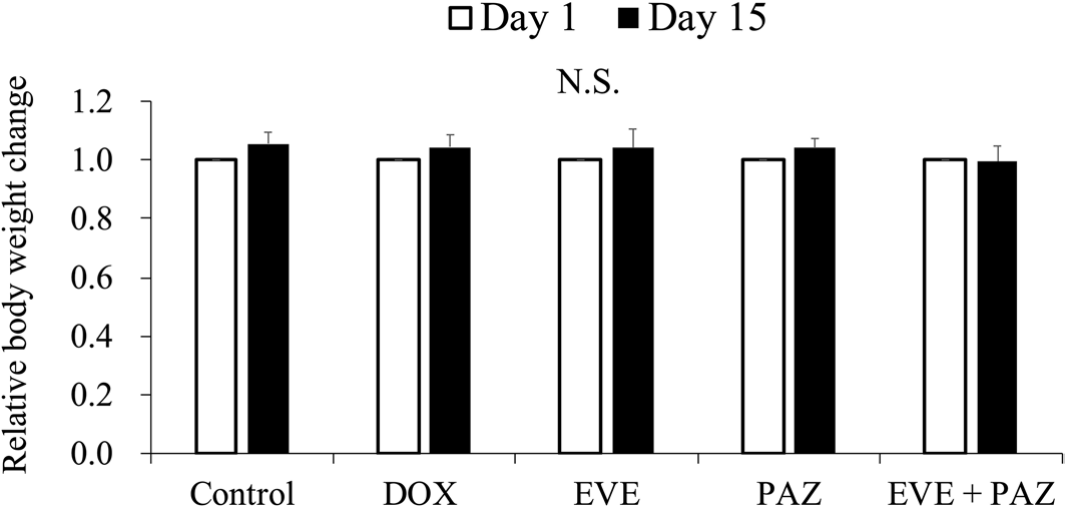
Efficacy of treatment on body weight in the osteosarcoma PDOX mouse model. The body-weight ratio of mice was calculated in each group on the 15th day from initial treatment relative to the body weight at the beginning of the experiment. Error bars show ± standard deviation. Statistical analysis was performed using the Tukey-Kramer test.

The combination of EVE and PAZ resulted in a slight body weight loss. However, no significant differences were observed among any group (Fig. 5).

### Efficacy of combination therapy of EVE and PAZ in the in vivo Gelfoam angiogenesis osteosarcoma patient-derived cell (OS-PDC) mouse model

The vessel length ratios at the end of the study were 1.44 ± 0.50 in Group 1 (untreated control); 1.22 ± 0.30 in Group 2 (DOX); 0.97 ± 0.31 in Group 3 (EVE); 0.80 ± 0.12 in Group 4 (PAZ); and 0.58 ± 0.08 in Group 5 (EVE and PAZ). The combination of EVE and PAZ group suppressed vessel length significantly compared with all other groups: (p = 0.012 compared to control; p = 0.008 compared to DOX; p = 0.008 compared to EVE; p = 0.017 compared to PAZ) (Figs. 7, 8).

**Figure 6:**
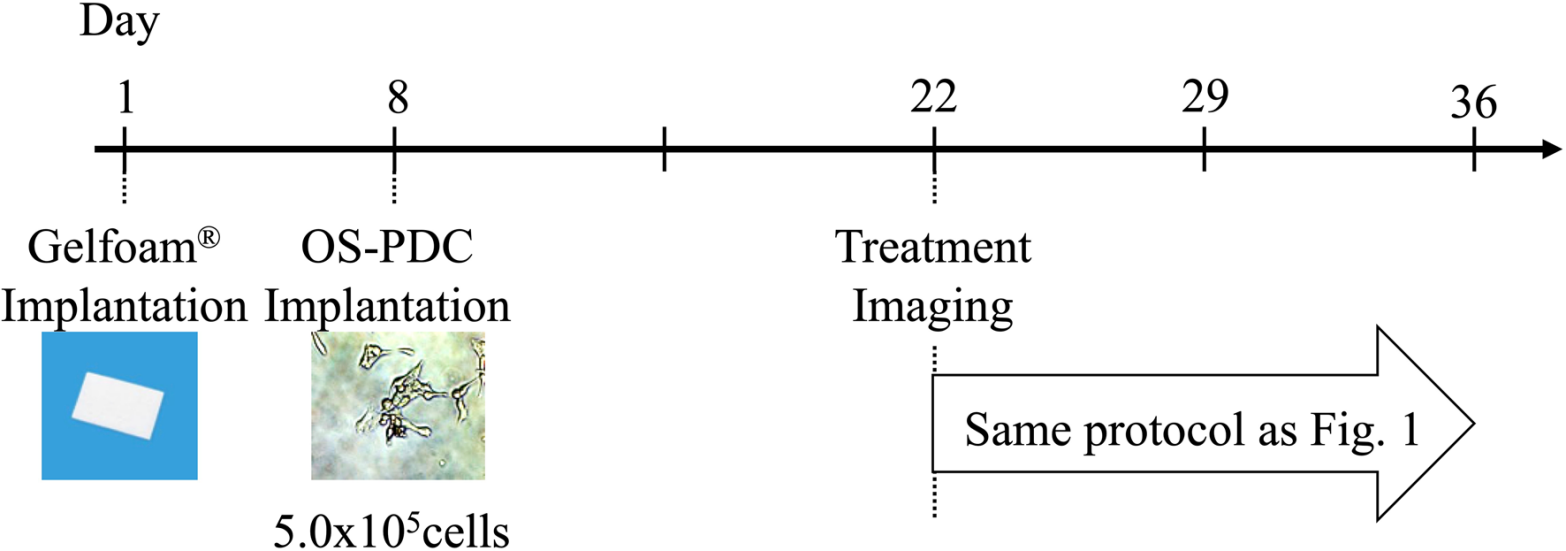
Treatment schema in in vivo Gelfoam angiogenesis assay model. Gelfoam saturated with βFGF was transplanted subcutaneously on the back of nestin-derived green fluorescent protein (ND-GFP) transgenic nude mice on day one. OS-PDCs were injected on day 8.

The therapeutic schedule for in vivo Gelfoam angiogenesis mouse model with OS-PDC is shown in Figs. 1 and 6. The treatment term was for 14 days.

**Figure 7:**
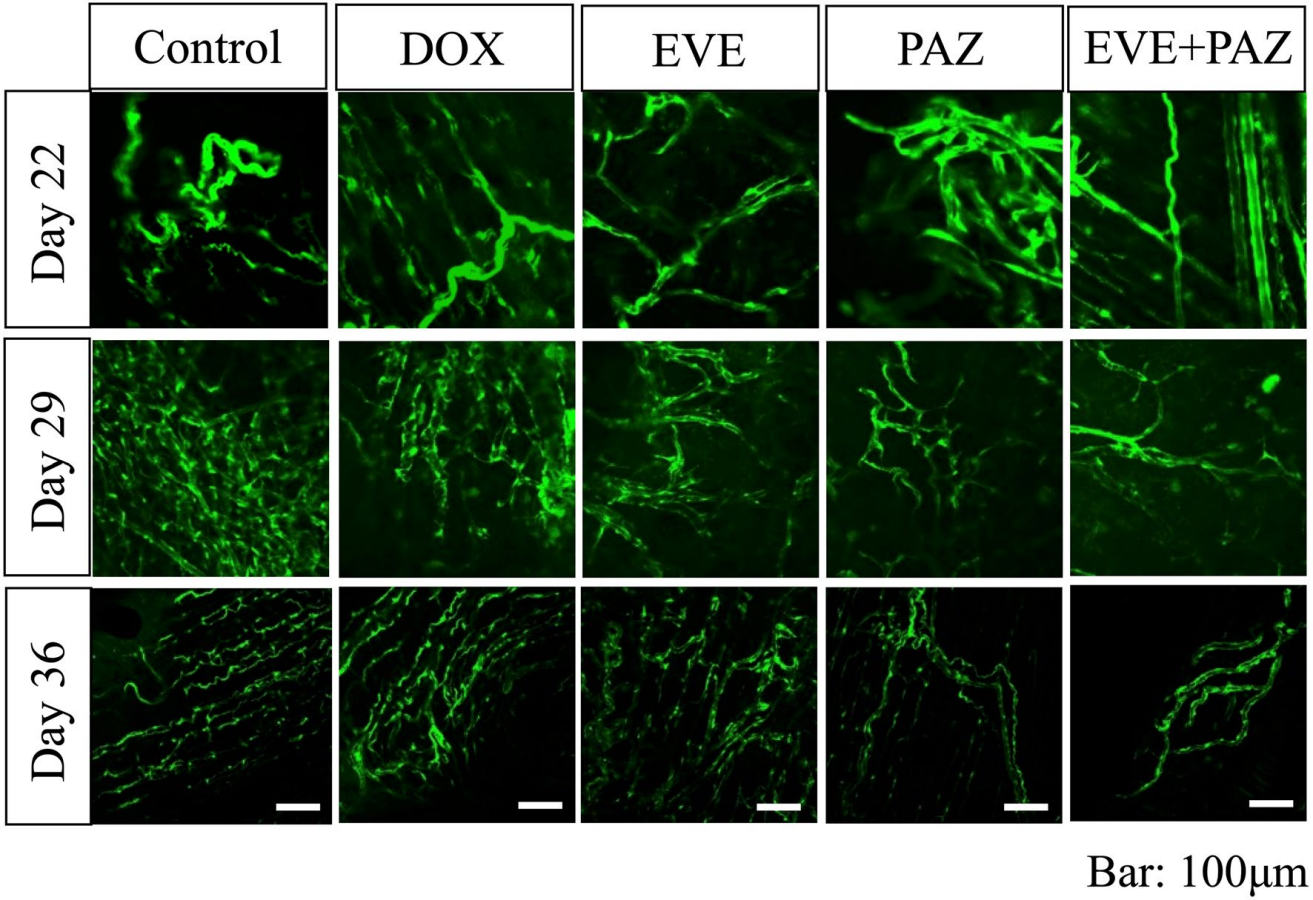
Images of ND-GFP-expressing new vessels in Gelfoam implanted with OS-PDCs in the in vivo Gelfoam angiogenesis assay. Images were obtained with the FV1000 confocal microscope. Scale bars 100 μm.

**Figure 8:**
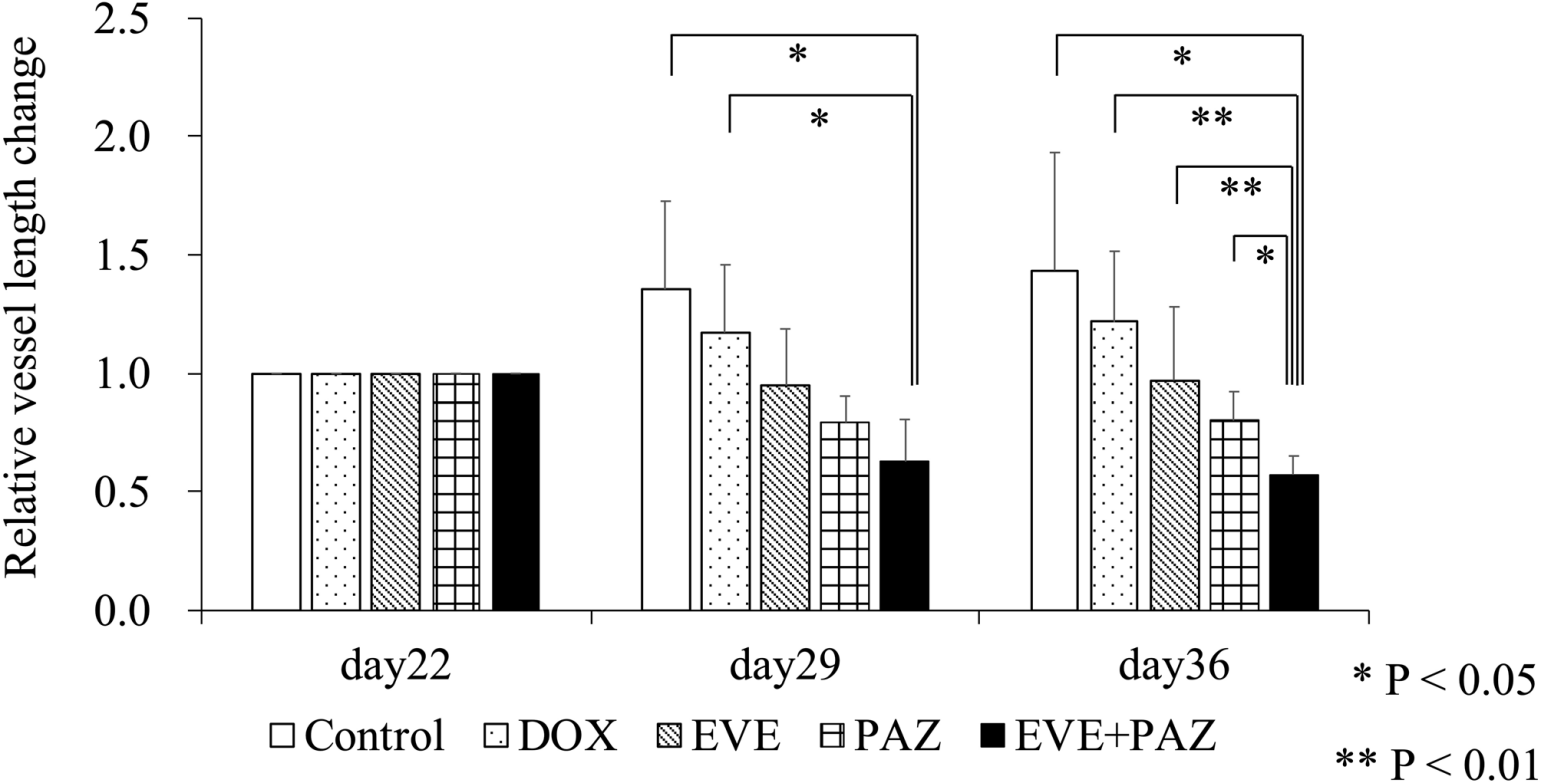
Drug efficacy in the in vivo Gelfoam angiogenesis assay. Bar graphs show the vessel length ratio on the day of measurement relative to day 22, which was the day of OS-PDC implantation into the Gelfoam previously inplanted in the mouse. Bar graphs express the average vessel length ratio and error bars show ± standard deviation. Statistical analysis was performed using Steel–Dwass test. *P < 0.05. **P < 0.01.

Representative vessel images obtained with the FV1000 confocal microscope in each group are shown in Fig. 7. The time-dependent change of the vessel length ratio is presented in Fig. 8.

## Discussion and conclusion

In the present study, the combination of EVE and PAZ demonstrated anti-tumor efficacy in the DOX-resistant lung-metastatic osteosarcoma PDOX mouse model compared with the untreated control, DOX, EVE-alone, and PAZ-alone. Moreover, the combination of EVE and PAZ had significant anti-angiogenesis efficacy in the in vivo Gelfoam angiogenesis OS-PDC mouse model compared to the untreated control and all other treatment.

EVE, an mTOR inhibitor, has anti-proliferative activity through inhibition of the PIK3/AKT/mTOR pathway and also has anti-angiogenesis activity by reducing production of hypoxia-inducible factor 1α and VEGF^5–8^. EVE has been used in the treatment of advanced breast and renal-cell cancer^3,4^. However, mTOR inhibitors have not shown efficacy against osteosarcoma clinically^40,41^. PAZ, a VEGFR inhibitor, inhibits the VEGF/VEGFR system, which diminishes new blood vessel formation^11–13^. Previous reports have shown that high expression of VEGF/VEGFR in osteosarcoma patients is a poor-outcome predictor^42–44^. Therefore, it is thought that both VEGF and VEGFR contribute to poor prognosis of osteosarcoma patients. PAZ had efficacy in some advanced cases of sarcoma^45,46^. However, there has not been a report that a VEGFR inhibitor could prolong overall survival and progression-free survival of osteosarcoma patients.

Blockade of angiogenesis in two different pathways with an mTOR inhibitor and a VEGFR inhibitor has been demonstrated to increase anti-tumor efficacy against several cancers in preclinical and clinical studies^15,47–49^. The combination of an mTOR and VEGFR inhibitor demonstrated 3.6 months progression free survival and 9.1 months overall survival in a urothelial carcinoma clinical trial^15^. In the present study, the efficacy of the mTOR and VEGFR inhibitor combination on osteosarcoma is the first report to the best of our knowledge.

We previously developed the in vivo Gelfoam angiogenesis mouse model that can image nascent vessel formation with GFP^29–32^. Our previous studies demonstrated strong induction of angiogenesis with an osteosarcoma cell line and anti-angiogenesis efficacy of tumor-targeting Salmonella typhimurium A1-R^31,32^. The present study indicated that combination of an mTOR and a VEGFR inhibitor reduced angiogenesis induced by the OS-PDC mouse model. The result of the present study also suggested that the anti-angiogenesis activity of the combination of mTOR and VEGFR inhibitors contributed to the efficacy on osteosarcoma tumor-growth inhibition.

Future gene-profiling studies will determine how the level of mTOR and VEGFR affect the response to the EVE-PAZ combination in the present and other PDOX models of osteosarcoma. Future experiments will also study the efficacy of the EVE-PAZ combination on the tumor micro-environment. The EVE-PAZ combination will also be studied for anti-metastatic efficacy and survival on the present PDOX model in future long-term experiments. Future experiments will also study the osteosarcoma cells that resisted DOX in the PDOX model to determine if they have increased DOX resistance and we will establish PDOX models from these super-DOX resistant osteosarcoma cells.

The results of the present study demonstrates that the combination of mTOR and VEGFR inhibitors is a promising therapeutic strategy for DOX-resistant recalcitrant metastatic osteosarcoma, in part due to strong anti-angiogenesis activity. Long-term treatment studies in additional osteosarcoma PDOX models will be necessary before clinical translation of the novel treatment strategy described in the present report. The osteosarcoma PDOX model mimiced the DOX resistant of the tumor in the patient.

## Materials and methods

### Mice

4–6 weeks old athymic *nu/nu* nude mice and nestin-regulatory-element-driven green fluorescent protein (ND-GFP) transgenic nude mice (AntiCancer Inc., San Diego, CA) were used in the present study for the PDOX and in vivo Gelfoam angiogenesis assay study, respectively. Mouse housing, imaging, and surgical procedures were performed as described in our previous publications^33–36^. The mice were humanely sacrificed as described in our previous publications^33–36^. An AntiCancer Institutional Animal Care and Use Committee (IACUC)-protocol was specifically approved for the present studies according to the procedures and principles summarized in the Guide for the Care and Use of Laboratory Animals, eighth edition, under the Public Health Service Approved Animal Welfare Assurance Number A3873-1^37^. The current study was carried out in compliance with the Animal Research: Reporting of Vivo Experiments (ARRIVE) guidelines.

### Patient-derived tumor

Informed consent from the patient and her parents was previously obtained under an Institutional Review Board-approved protocol of University of California, Los Angels (UCLA) (IRB #10-001857)^38^. A 16-year-old female with high-grade osteosarcoma of left-distal-femur received neoadjuvant chemotherapy with DOX and had surgery with replacement of the distal femur. One year after surgery, three bilateral metachronous pulmonary metastases recurred, which were resected at UCLA. The resected tumor was previously established as an osteosarcoma PDOX mouse model^38^. All methods performed with the patient-derived tumor were performed with the relevant guidelines and regulations.

### Establishment of the osteosarcoma PDOX mouse model by surgical orthotopic implantation (SOI)

The osteosarcoma tumor from the lung metastasis was previously initially implanted on the subcutaneous back space of nude mice to establish the tumor^38^. The subcutaneously-grown tumors were excised and 9 mm^3^ small fragments were made from excised tumor. After mice were put under anesthesia using a ketamine solution, a 10-mm skin incision was made on the lateral side of right knee. The vastus lateralis muscle was cut and divided to expose the lateral condyle of the distal femur. The lateral condyle of distal femur was cut with a scissors to make a cavity for implantation of the tumor fragment. A single 9 mm^3^ tumor fragment was orthotopically implanted the resected cavity of the lateral condyle in order to establish a PDOX mouse model. The wounds in the muscle and skin were sutured as described in our previous publication^38^.

### Therapeutic study design in the osteosarcoma PDOX mouse model

Five groups of seven mice were randomly assigned from the osteosarcoma PDOX mouse models as follows: Group 1, control with PBS, i.p. (i.p., n = 7); Group 2, DOX alone (3 mg/kg, i.p., weekly for 2 weeks, n = 7); Group 3, EVE alone (5 mg/kg, oral gavage, daily for 2 weeks, n = 7); Group 4, PAZ alone (50 mg/kg, oral gavage, daily for 2 weeks, n = 7); Group 5, combination of EVE and PAZ (EVE: 5 mg/kg, PAZ: 50 mg/kg, n = 7) (Fig. 1). Treatment was started 21 days after orthotopic implantation of the tumor. All mice were sacrificed on day 15 after treatment initiation.

The long and short diameters of tumors were monitored with calipers and body weight was monitored with a digital weight scale twice a week. The tumor volume was calculated using the following formula: Tumor volume (mm^3^) = long diameter (mm) x short diameter (mm) x short diameter (mm) × 1/2. The tumor volume ratio was equal to the tumor volume at each time point divided to the initial time point.

### Histopathology

Resected tumor specimens were placed in 10% formalin for fixation. The tumor specimens were embedded in melted paraffin before sectioning. Tissue sections (5 μm) were rehydrated using ethanol and deparaffinized using xylene. Hematoxylin and eosin (H&E) staining was conducted with standard protocols^27,32^.

### Implantation of Gelfoam

ND-GFP transgenic nude mice were put under anesthesia with the ketamine solution. Basic fibroblast growth factor (βFGF; Millipore, Billerica, MA, U.S.A., 300 ng) and RPMI-1640 medium (75 μl) were added to Gelfoam blocks (5 × 5 mm). The treated Gelfoam was then implanted in the subcutaneous space of ND-GFP transgenic nude mice^29–31^.

### Primary cell culture of OS-PDCs

OS-PDCs were established as follows: tumors were minced mechanically with scissors and seeded in DMEM (Sigma-Aldrich, St. Louis, MO) with 10% fetal bovine serum (GIBCO, Grand Island, NY), 100 U penicillin G, and 100 μg/ml streptomycin (Gibco) in 100 mm plastic dishes. The minced tumors with medium were cultured at 37 °C, in a 5% CO_2_ incubator. 24 h after seeding, medium was changed to remove loose debris and unattached cells.

### Injection of OS-PDCs into mouse-implanted Gelfoam

Seven days after implantation of Gelfoam, OS-PDCs (5 × 10^5^ cells) were injected into the previously implanted Gelfoam in ND-GFP transgenic nude mice which express GFP in nascent blood vessels. ND-GFP transgenic nude mice were put under anesthesia with the ketamine solution. A skin incision was made on the back from the shoulder to the buttock. The subcutaneous tissue was exfoliated to make a skin flap without damaging the vessels. The skin flap was unfolded and immobilized on a flat stand. A 6–0 nylon suture was used to suture the skin after imaging^30,31,39^.

### Imaging and measurement of fluorescent vessel length

Skin flaps were made for imaging at 22, 29, and 36 days after implantation of Gelfoam. The FV1000 confocal microscope with an XLUMPLFLx20x (0.95 numerical aperture (NA)) water immersion objective (Olympus Corp., Tokyo, Japan) was used to visualize GFP-expressing blood vessels. GFP had a peak excitation at 488 nm with an argon laser. FV10-ASW Fluoview software (Olympus) was used to display image. Measurement of the vessel length was conducted with FV10-ASW Fluoview software. A 6–0 nylon suture was used to suture the skin after imaging^31^.

### Therapeutic study design in the in vivo Gelfoam fluorescence angiogenesis mouse model with OS-PDCs

In vivo Gelfoam fluorescence angiogenesis mouse models were randomly assigned into the control and four-therapeutic groups the same as the PDOX model groups described above (Fig. 6). Imaging of vessel length measurements started on day-22, which is 14 days after implantation of OS-PDCs into the Gelfoam (Fig. 2). The vessel-length ratio was determined on the vessel length at any given time point relative to the vessel length on day 22. For each mouse, eight fields were selected to measure vessel length.

### Statistical analysis

JMP version 13.0 (SAS Institute Japan, Tokyo, Japan) was employed for statistical analyses. The Steel–Dwass test was used to analyze the tumor volume ratio and vessel length ratio, and the Tukey-Kramer test was used to analyze body weight ratio. Line graphs or bar graphs show the mean data, and error bars show ± standard deviation. A probability value of p < 0.05 was determined to have significant difference.

## References

[1] Isakoff MS, Bielack SS, Meltzer P, Gorlick R (2015). Osteosarcoma: Current treatment and a collaborative pathway to success. J. Clin. Oncol..

[2] Seto T, Song MN, Trieu M, Yu J, Sidhu M, Liu CM (2019). Real-world experiences with pazopanib in patients with advanced soft tissue and bone sarcoma in Northern California. Med. Sci. (Basel)..

[3] Motzer RJ, Escudier B, Oudard S, Hutson TE, Porta C, Bracarda S (2008). Efficacy of everolimus in advanced renal cell carcinoma: A double-blind, randomised, placebo-controlled phase III trial. Lancet.

[4] Yardley DA, Noguchi S, Pritchard KI, Burris HA, Baselga J, Gnant M (2013). Everolimus plus exemestane in postmenopausal patients with HR(+) breast cancer: BOLERO-2 final progression-free survival analysis. Adv. Ther..

[5] Mabuchi S, Altomare DA, Cheung M, Zhang L, Poulikakos PI, Hensley HH (2007). RAD001 inhibits human ovarian cancer cell proliferation, enhances cisplatin-induced apoptosis, and prolongs survival in an ovarian cancer model. Clin. Cancer Res..

[6] Dormond O, Madsen JC, Briscoe DM (2007). The effects of mTOR-Akt interactions on anti-apoptotic signaling in vascular endothelial cells. J Biol Chem..

[7] Falcon BL, Barr S, Gokhale PC, Chou J, Fogarty J, Depeille P (2011). Reduced VEGF production, angiogenesis, and vascular regrowth contribute to the antitumor properties of dual mTORC1/mTORC2 inhibitors. Cancer Res..

[8] Faes S, Santoro T, Demartines N, Dormond O (2017). Evolving significance and future relevance of anti-angiogenic activity of mTOR inhibitors in cancer therapy. Cancers (Basel)..

[9] Rini BI (2007). Vascular endothelial growth factor-targeted therapy in renal cell carcinoma: Current status and future directions. Clin. Cancer Res..

[10] van der Graaf WT, Blay JY, Chawla SP, Kim DW, Bui-Nguyen B, Casali PG (2012). Pazopanib for metastatic soft-tissue sarcoma (PALETTE): A randomised, double-blind, placebo-controlled phase 3 trial. Lancet.

[11] McMahon G (2000). VEGF receptor signaling in tumor angiogenesis. Oncologist..

[12] Gotink KJ, Verheul HM (2010). Anti-angiogenic tyrosine kinase inhibitors: What is their mechanism of action?. Angiogenesis.

[13] Shibuya M (2013). Vascular endothelial growth factor and its receptor system: Physiological functions in angiogenesis and pathological roles in various diseases. J. Biochem..

[14] Pignochino Y, Dell'Aglio C, Inghilleri S, Zorzetto M, Basiricò M, Capozzi F (2015). The combination of sorafenib and everolimus shows antitumor activity in preclinical models of malignant pleural mesothelioma. BMC Cancer.

[15] Bellmunt J, Lalani AA, Jacobus S, Wankowicz SA, Polacek L, Takeda DY (2018). Everolimus and pazopanib (E/P) benefit genomically selected patients with metastatic urothelial carcinoma. Br. J. Cancer..

[16] Fu X, Guadagni F, Hoffman RM (1992). A metastatic nude-mouse model of human pancreatic cancer constructed orthotopically with histologically intact patient specimens. Proc. Natl. Acad. Sci. USA.

[17] Fu X, Hoffman RM (1993). Human ovarian carcinoma metastatic models constructed in nude mice by orthotopic transplantation of histologically-intact patient specimens. Anticancer Res..

[18] Metildi CA, Kaushal S, Luiken GA, Talamini MA, Hoffman RM, Bouvet M (2014). Fluorescently labeled chimeric anti-CEA antibody improves detection and resection of human colon cancer in a patient-derived orthotopic xenograft (PDOX) nude mouse model. J. Surg. Oncol..

[19] Hiroshima Y, Zhang Y, Zhang N, Maawy A, Mii S, Yamamoto M (2015). Establishment of a patient-derived orthotopic Xenograft (PDOX) model of HER-2-positive cervical cancer expressing the clinical metastatic pattern. PLoS ONE.

[20] Yamamoto M, Zhao M, Hiroshima Y, Zhang Y, Shurell E, Eilber FC (2016). Efficacy of tumor-targeting salmonella A1-R on a melanoma patient-derived orthotopic xenograft (PDOX) nude-mouse model. PLoS ONE.

[21] Kawaguchi K, Igarashi K, Murakami T, Kiyuna T, Zhao M, Zhang Y (2017). Salmonella typhimurium A1-R targeting of a chemotherapy-resistant BRAF-V600E melanoma in a patient-derived orthotopic xenograft (PDOX) model is enhanced in combination with either vemurafenib or temozolomide. Cell Cycle.

[22] Oshiro H, Tome Y, Kiyuna T, Yoon SN, Lwin TM, Han Q (2019). Oral recombinant methioninase overcomes colorectal-cancer liver metastasis resistance to the combination of 5-fluorouracil and oxaliplatinum in a patient-derived orthotopic xenograft mouse model. Anticancer Res..

[23] Murakami T, DeLong J, Eilber FC, Zhao M, Zhang Y, Zhang N (2016). Tumor-targeting Salmonella typhimurium A1-R in combination with doxorubicin eradicate soft tissue sarcoma in a patient-derived orthotopic xenograft (PDOX) model. Oncotarget.

[24] Kiyuna T, Murakami T, Tome Y, Igarashi K, Kawaguchi K, Russell T (2017). Labeling the stroma of a patient-derived orthotopic xenograft (PDOX) mouse model of undifferentiated pleomorphic soft-tissue sarcoma with red fluorescent protein for rapid non-invasive imaging for drug screening. J. Cell Biochem..

[25] Miyake, K. The combination of temozolomide-irinotecan regresses a doxorubicin-resistant patient-derived orthotopic xenograft (PDOX) nude-mouse model of recurrent Ewing’s sarcoma with a FUS-ERG fusion and CDKN2A deletion: Direction for third-line patient therapy (2017). 10.18632/oncotarget.20789 PMC5732717 29262551

[26] Igarashi K, Kawaguchi K, Kiyuna T, Miyake K, Miyake M, Li Y (2018). Temozolomide combined with irinotecan regresses a cisplatinum-resistant relapsed osteosarcoma in a patient-derived orthotopic xenograft (PDOX) precision-oncology mouse model. Oncotarget.

[27] Oshiro H, Tome Y, Kiyuna T, Miyake K, Kawaguchi K, Higuchi T (2019). Temozolomide targets and arrests a doxorubicin-resistant follicular dendritic-cell sarcoma patient-derived orthotopic xenograft mouse model. Tissue Cell..

[28] Hoffman RM (2015). Patient-derived orthotopic xenografts: better mimic of metastasis than subcutaneous xenografts. Nat. Rev. Cancer..

[29] Amoh Y, Yang M, Li L, Reynoso J, Bouvet M, Moossa AR (2005). Nestin-linked green fluorescent protein transgenic nude mouse for imaging human tumor angiogenesis. Cancer Res..

[30] Amoh Y, Li L, Katsuoka K, Bouvet M, Hoffman RM (2007). GFP-expressing vascularization of Gelfoam as a rapid in vivo assay of angiogenesis stimulators and inhibitors. Biotechniques.

[31] Uehara F, Tome Y, Miwa S, Hiroshima Y, Yano S, Yamamoto M (2014). Osteosarcoma cells enhance angiogenesis visualized by color-coded imaging in the in vivo Gelfoam assay. J. Cell Biochem..

[32] Kiyuna T, Tome Y, Uehara F, Murakami T, Zhang Y, Zhao M (2018). Tumor-targeting Salmonella typhimurium A1-R inhibits osteosarcoma angiogenesis in the in vivo gelfoam assay visualized by color-coded imaging. Anticancer Res..

[33] Igarashi K, Kawaguchi K, Kiyuna T, Miyake K, Miyaki M, Yamamoto N (2018). Metabolic targeting with recombinant methioninase combined with palbociclib regresses a doxorubicin-resistant dedifferentiated liposarcoma. Biochem. Biophys. Res. Commun..

[34] Miyake K, Kiyuna T, Miyake M, Zhao M, Wangsiricharoen S, Kawaguchi K (2018). Tumor-targeting Salmonella typhimurium A1-R overcomes partial carboplatinum-resistance of a cancer of unknown primary (CUP). Tissue Cell..

[35] Kawaguchi K, Higuchi T, Li S, Han Q, Tan Y, Igarashi K (2018). Combination therapy of tumor-targeting Salmonella typhimurium A1-R and oral recombinant methioninase regresses a BRAF-V600E-negative melanoma. Biochem. Biophys. Res. Commun..

[36] Higuchi T, Kawaguchi K, Miyake K, Han Q, Tan Y, Oshiro H (2018). Oral recombinant methioninase combined with caffeine and doxorubicin induced regression of a doxorubicin-resistant synovial sarcoma in a PDOX mouse model. Anticancer Res..

[37] Kiyuna T, Tome Y, Murakami T, Miyake K, Igarashi K, Kawaguchi K (2018). A combination of irinotecan/cisplatinum and irinotecan/temozolomide or tumor-targeting Salmonella typhimurium A1-R arrest doxorubicin- and temozolomide-resistant myxofibrosarcoma in a PDOX mouse model. Biochem. Biophys. Res. Commun..

[38] Murakami T, Igarashi K, Kawaguchi K, Kiyuna T, Zhang Y, Zhao M (2017). Tumor-targeting Salmonella typhimurium A1-R regresses an osteosarcoma in a patient-derived xenograft model resistant to a molecular-targeting drug. Oncotarget.

[39] Amoh Y, Li L, Tsuji K, Moossa AR, Katsuoka K, Hoffman RM (2006). Dual-color imaging of nascent blood vessels vascularizing pancreatic cancer in an orthotopic model demonstrates antiangiogenesis efficacy of gemcitabine. J. Surg. Res..

[40] Grignani G, Palmerini E, Ferraresi V, D'Ambrosio L, Bertulli R, Asaftei SD (2015). Sorafenib and everolimus for patients with unresectable high-grade osteosarcoma progressing after standard treatment: A non-randomised phase 2 clinical trial. Lancet Oncol..

[41] Martin-Broto J, Redondo A, Valverde C, Vaz MA, Mora J, Garcia Del Muro X (2017). Gemcitabine plus sirolimus for relapsed and progressing osteosarcoma patients after standard chemotherapy: A multicenter, single-arm phase II trial of Spanish Group for Research on Sarcoma (GEIS). Ann. Oncol..

[42] Hassan SE, Bekarev M, Kim MY, Lin J, Piperdi S, Gorlick R (2012). Cell surface receptor expression patterns in osteosarcoma. Cancer.

[43] Ohba T, Cates JM, Cole HA, Slosky DA, Haro H, Ando T (2014). Autocrine VEGF/VEGFR1 signaling in a subpopulation of cells associates with aggressive osteosarcoma. Mol. Cancer Res..

[44] Liu K, Ren T, Huang Y, Sun K, Bao X, Wang S (2017). Apatinib promotes autophagy and apoptosis through VEGFR2/STAT3/BCL-2 signaling in osteosarcoma. Cell Death Dis..

[45] Longhi A, Paioli A, Palmerini E, Cesari M, Abate ME, Setola E (2019). Pazopanib in relapsed osteosarcoma patients: Report on 15 cases. Acta Oncol..

[46] Aggerholm-Pedersen N, Rossen P, Rose H, Safwat A (2020). Pazopanib in the treatment of bone sarcomas: Clinical experience. Transl Oncol..

[47] Ikezoe T, Yang Y, Nishioka C, Bandobashi K, Nakatani H, Taguchi T (2006). Effect of SU11248 on gastrointestinal stromal tumor-T1 cells: enhancement of growth inhibition via inhibition of 3-kinase/Akt/mammalian target of rapamycin signaling. Cancer Sci..

[48] Juengel E, Engler J, Natsheh I, Jones J, Mickuckyte A, Hudak L (2009). Combining the receptor tyrosine kinase inhibitor AEE788 and the mammalian target of rapamycin (mTOR) inhibitor RAD001 strongly inhibits adhesion and growth of renal cell carcinoma cells. BMC Cancer.

[49] Park HS, Hong SK, Oh MM, Yoon CY, Jeong SJ, Byun SS (2014). Synergistic antitumor effect of NVP-BEZ235 and sunitinib on docetaxel-resistant human castration-resistant prostate cancer cells. Anticancer Res..

